# Reference curves for trabecular bone score adjusted for soft tissue thickness in children and adolescents from Mexico City

**DOI:** 10.1007/s11657-025-01595-4

**Published:** 2025-08-06

**Authors:** Miguel Angel Guagnelli, Desiree Lopez-Gonzalez, Karen Hind, Enisa Shevroja, Didier Hans, Patricia Clark

**Affiliations:** 1 Clinical Epidemiology Unit, Hospital Infantil de México Federico Gomez – Facultad de Medicina, Universidad Nacional Autónoma de México, Mexico City, Mexico; 2 Medimaps group SA, Plan-les-Ouates, Geneva, Switzerland; 3 Lancaster Medical School, University of Lancaster, Lancaster, UK; 4 Wolfson Institute for Health and Wellbeing, Durham University, Durham, UK; 5 Interdisciplinary Centre of Bone Diseases, Bone and Joint Department, Lausanne University Hospital (CHUV) & University of Lausanne, Lausanne, Switzerland

**Keywords:** Trabecular bone score (TBS), Pediatrics, Puberty, Reference curves

## Abstract

***Summary*:**

This study proposes age- and sex-specific trabecular bone score (TBS) reference curves for Mexican children and adolescents. Using the latest software version, results highlight significant pubertal changes and provide reference data for assessing pediatric bone health, paving the way for a wider use of this technology in children and adolescents.

**Purpose:**

Trabecular Bone Score (TBS) is a grey scale texture measure that correlates with bone microarchitecture derived from dual-energy X-ray absorptiometry (DXA). While extensively studied in adults, limited data exist for pediatric populations. This study aims to develop age- and sex-specific reference curves for TBS adjusted for abdominal soft tissue thickness in healthy children and adolescents from Mexico City.

**Methods:**

This cross-sectional study reanalyzed data from 1552 healthy participants (5–18 years) who underwent lumbar spine DXA scans using Lunar iDXA and TBS iNsight 4.0 (Core Module 19.4.0), which accounts for soft tissue thickness. Generalized Additive Models for Location, Scale, and Shape (GAMLSS) were employed to construct smoothed percentile curves. TBS values were stratified by age, sex, and Tanner stage, with descriptive statistics and outlier exclusions.

**Results:**

TBS showed distinct age- and sex-related trajectories, with steep increases during puberty. Girls demonstrated a sharper rise in TBS starting at age 9, peaking by age 16, while boys exhibited a more gradual increase starting at age 10–11, peaking by age 18. Differences were also observed between Tanner stages, with the most significant changes occurring from stages 2 to 3.

**Conclusion:**

This study proposes the first TBS reference curves for Mexican children and adolescents using the latest software version. This data may prove to be a valuable tool for assessing bone health in pediatric populations. Yet further research to explore TBS’s utility in predicting bone fragility in pediatric population as well as its life-course trends.

**Supplementary Information:**

The online version contains supplementary material available at 10.1007/s11657-025-01595-4.

## Introduction

Trabecular bone score (TBS) is a validated measurement of bone microarchitecture that provides information about the quality of bone that is not captured by conventional areal bone mineral density (aBMD) measurements. It is derived from standard high-resolution dual-energy X-ray absorptiometry (DXA) images and is based on the texture analysis of the bone image using an experimental variogram. TBS is a valuable tool to predict fracture risk independently of aBMD [1].

It has been theorized that TBS could provide additional information about bone health in children and adolescents beyond aBMD measurements. TBS has also been used to evaluate bone health in children and adolescents, and it appears to be a useful tool for such purposes in the pediatric age. However, few studies have been published in healthy children [2–5]. So far TBS has been used to assess bone health in children with various medical conditions that affect bone health, including anorexia nervosa [6], inflammatory bowel disease [7], cerebral palsy [8], end-stage renal disease [9], and osteogenesis imperfecta [10], but only in an exploratory manner. Therefore, despite its potential as a promising tool for evaluating bone health for children and adolescents, there is a paucity of studies in the pediatric age group.

Our group previously published preliminary data evaluating bone age as a relevant factor in assessing TBS in healthy children, finding that increases in TBS during puberty in boys and girls are more closely related to bone age than chronological age [11]. Recent advancements to the TBS algorithm have further improved reliability by considering abdominal thickness [12], which previously caused a certain degree of variability in children, a factor that has been accounted for in the most recent version of the software and therefore renders it ready for better evaluation of pediatric population.

However, before TBS can be routinely used in pediatric care, two essential prerequisites need to be covered. First, as it was done in early studies when DXA first appeared in the early nineties and from which the concept of Peak Bone Mass was derived, it is essential to lay out the trajectories with which TBS values evolve with time considering variations by age, gender, the impact of puberty, and lifestyle variables. The current International Society for Clinical Densitometry (ISCD) position statement on TBS states that studies show there are @few studies demonstrating relationships between TBS and fracture risk in adults aged 20–39 years [13], although TBS has been found to be significantly lower in young patients with secondary osteoporosis and predictive of vertebral fracture [13–16].

Second, variations in reference aBMD values by country and ethnic group are recognized and, although ISDC recommends using a uniform non-race adjusted normative database to calculate T-score in adult women and men, it also states that if local reference data are available they should be used to calculate *Z*-scores [17]. It is yet unknown if this same principle applies to TBS in children, but the common approach to the subject is that every population should have their own set of reference values according to sex and age in children.

The potential of TBS to offer insights into bone health in children, beyond what is measured by traditional DXA assessments like aBMD and bone mineral apparent density (BMAD), is not yet fully understood. Establishing reference values for TBS in this demographic is a critical step and using TBS version 4.0 which includes an adjustment for tissue thickness. These benchmarks not only enhance the accuracy of bone health assessments in young populations but also pave the way for further studies. Such research could explore TBS’s effectiveness in predicting fracture risk and its utility in monitoring bone health, laying the groundwork for future investigations into fracture prediction among children and adolescents.

To our best knowledge, there are two published studies including TBS reference data for children and adolescents. The first and larger one comes from the Bone Mineral Density in Childhood Study, where bone measurements were obtained from 2014 children and adolescents at five clinical centers in the USA [4]. The second one is from Brazil where the authors included 349 children [5]. However, in the latter the researchers used an earlier version of TBS which is not recommended for use in children. Therefore, the present study aims to develop reference TBS data, using TBS version 4.0 (core module version 19.4.0) for Mexican children and adolescents and add to the body of knowledge needed to start solving questions. Given the paucity of pediatric data, we intend to build specific data and graphs to describe TBS for our pediatric population.

## Patients and methods

### Population

Data for this study is a reanalysis of images obtained for the study “Reference values for body composition in healthy urban Mexican children and adolescents” HIM 2015–055 [18] which was conducted as a population-based, cross-sectional investigation on healthy children and adolescents. Previously published information provided a thorough account of the study’s design, participants, procedures, and inclusion/exclusion criteria. In brief, a multistage random sampling method was used to invite children and adolescents from Mexico City and its surrounding areas to participate in the study between March 2015 and November 2019. The participants underwent clinical assessments and anthropometric measurements. Tanner staging was performed through clinical examination by trained pediatricians who received standardized instruction based on the Marshall and Tanner criteria. Blood samples were obtained after an overnight fast to measure various factors such as serum glucose, insulin, triglycerides, total cholesterol, LDL, and HDL. Participants were excluded if they had abnormal fasting glucose, triglycerides, total cholesterol, HDL, or LDL levels outside reference values for age and sex. The primary aim was to include clinically healthy children and adolescents and exclude individuals with metabolic abnormalities from the study (i.e. dyslipidemia, insulin resistance) and not solely on the basis of BMI. Those with conditions or using medications that may alter bone metabolism, such as endocrine disorders, chronic inflammatory diseases, or any treatment known to impact bone health were also excluded. Although 2104 children and adolescents aged 4.5 to 20 years were recruited for the original study, only 1552 (those aged 5 to 18) were included. In this analysis, we follow the same premise.

### DXA measurements

The participants underwent whole-body and postero-anterior lumbar spine (L1–L4) scans, which were conducted using the Lunar-iDXA device (GE Healthcare, Madison, WI using Encore V15) and calibrated daily using a calibration phantom according to the manufacturers’ recommendations. The subject positioning for the scans was based on the manufacturer’s instructions, and the scans followed the recommendations of the ISCD and were performed by certified personnel [19].

### TBS evaluation

We retrieved DXA images and sent them to an expert from the Interdisciplinary Center for Bone Diseases (Lausanne, Switzerland) to conduct a blind analysis with the pre-release version 4.0 (Core Module version 19.4.0) of the TBS iNsight software (MedImaps, Geneva, Switzerland). This version of TBS iNsight introduces an advanced algorithm that accounts for the thickness of abdominal soft tissue, as measured from lumbar spine DXA scans. The algorithm’s adjustments were designed to cover soft tissue thicknesses ranging from 6.5 to 32.5 cm, ensuring it suits children and adults. By incorporating corrections for abdominal soft tissue thickness, we directly address and mitigate the previously observed negative correlation between TBS values and BMI, significantly enhancing the tool’s accuracy and relevance for broader applications [12]. The software’s TBS values as output are a series of unitless values that include individual L1–L4 TBS values and a mean of them labeled as mean TBS. For the data analysis, we used mean TBS to calculate the data included in tables and used to build the curves.

## Results

**Fig. 1:**
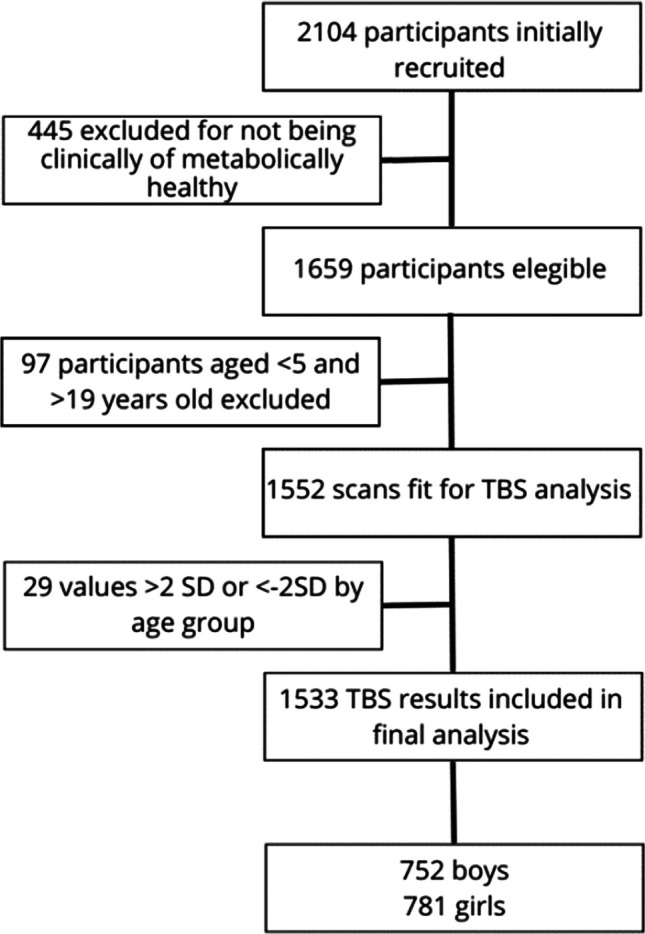
Flowchart of participants

In our study to calculate reference values, we identified 1659 children and adolescents, aged 4.5 to 20 years, as clinically and metabolically healthy, making them suitable candidates for generating reference values from their DXA scans. We evaluated the original DXA scans and found 1552 scans valid for TBS analysis. Valid scans were defined as those with adequate image quality and spine region segmentation allowing reliable TBS computation. Scans were excluded if they had artifacts, vertebral anomalies, or incomplete lumbar imaging. This subset included 781 females (50.9%) and 752 males (49.1%). Although we considered all participants for the Tanner group analysis, we only included those aged 5 to 18 in the reference tables and graphs. Figure 1 illustrates the selection process through a flowchart. Anthropometric characteristics are shown in Table 1. TBS values summarized by age group, from 5 to 18 years are shown in Table 2.

**Table 1:** Anthropometric characteristics by age group and sex. Age bands represent full-year intervals (e.g., 5.0 to 5.99 years)

Age	Males				Females			
	*n*	Weight (kg)	Height (cm)	BMI (kg/m^2^)	*n*	Weight (kg)	Height (cm)	BMI (kg/m^2^)
5	49	20.3 ± 6.2	111.8 ± 9.2	16.0 ± 2.4	62	19.7 ± 4.3	110.5 ± 6.7	16.0 ± 2.6
6	50	22.2 ± 4.5	117.7 ± 4.9	15.9 ± 2.1	56	21.1 ± 3.6	115.5 ± 5.6	15.8 ± 1.9
7	82	28.3 ± 6.1	128.4 ± 6.5	15.9 ± 2.1	62	27.4 ± 6.6	128.2 ± 6.7	16.5 ± 2.7
8	76	28.3 ± 6.1	128.4 ± 6.5	17.0 ± 2.7	69	27.4 ± 6.6	128.2 ± 6.7	16.5 ± 2.7
9	63	31.7 ± 8.2	135.5 ± 9.1	18.3 ± 2.9	48	29.8 ± 5.7	132.1 ± 5.7	17.0 ± 2.3
10	54	35.4 ± 7.6	138.5 ± 8.7	18.3 ± 2.9	63	35.1 ± 7.7	139.4 ± 7.3	18.3 ± 3.0
11	55	40.3 ± 9.2	146.2 ± 8.1	19.7 ± 3.5	76	39.6 ± 7.6	145.3 ± 7.2	18.6 ± 3.5
12	50	43.7 ± 11.5	152.2 ± 9.6	18.6 ± 3.5	40	45.3 ± 9.8	151.3 ± 9.3	19.7 ± 3.5
13	47	48.1 ± 9.1	158.1 ± 7.2	19.1 ± 3.2	54	49.5 ± 6.8	153.9 ± 6.5	20.8 ± 3.5
14	49	55.0 ± 10.2	165.0 ± 7.2	20.2 ± 3.0	50	50.0 ± 7.5	155.9 ± 9.1	20.8 ± 2.8
15	59	57.3 ± 10.2	167.8 ± 7.8	20.7 ± 2.8	59	53.7 ± 8.0	158.4 ± 7.0	21.2 ± 2.9
16	47	59.5 ± 9.1	169.4 ± 5.3	20.7 ± 2.9	50	56.8 ± 7.0	158.4 ± 7.0	20.7 ± 2.9
17	34	60.6 ± 5.7	170.6 ± 5.7	20.8 ± 2.4	31	55.4 ± 7.5	158.8 ± 5.7	21.9 ± 2.7
18	33	62.2 ± 9.9	170.8 ± 6.4	21.2 ± 2.7	31	55.4 ± 8.6	158.8 ± 5.7	21.9 ± 2.7

**Table 2:** TBS version 4.0 (core module version 19.4.0) values by sex and age group. including mean and ±2SD. Age bands represent full-year intervals (e.g., 5.0 to 5.99 years)

Age	Mean TBS	SD	Z-scores				
			−2	−1	0	1	2
Females							
5	1.200	0.075	1.051	1.125	1.200	1.275	1.349
6	1.219	0.066	1.067	1.133	1.200	1.266	1.332
7	1.200	0.069	1.067	1.133	1.200	1.266	1.332
8	1.190	0.07	1.05	1.12	1.19	1.26	1.33
9	1.205	0.08	1.05	1.13	1.21	1.28	1.36
10	1.197	0.07	1.07	1.13	1.2	1.26	1.33
11	1.244	0.08	1.08	1.16	1.24	1.33	1.41
12	1.304	0.08	1.14	1.22	1.3	1.39	1.47
13	1.338	0.08	1.17	1.26	1.34	1.42	1.5
14	1.356	0.07	1.21	1.29	1.36	1.43	1.5
15	1.378	0.06	1.26	1.32	1.38	1.44	1.5
16	1.386	0.06	1.26	1.32	1.39	1.45	1.51
17	1.397	0.058	1.281	1.339	1.397	1.455	1.513
18	1.413	0.062	1.288	1.350	1.413	1.475	1.537
Males							
5	1.221	0.060	1.100	1.161	1.221	1.281	1.342
6	1.22	0.07	1.09	1.15	1.22	1.29	1.36
7	1.21	0.07	1.08	1.14	1.21	1.28	1.34
8	1.22	0.07	1.08	1.15	1.22	1.29	1.36
9	1.21	0.07	1.07	1.14	1.21	1.28	1.35
10	1.21	0.06	1.09	1.15	1.21	1.27	1.33
11	1.22	0.05	1.11	1.17	1.22	1.28	1.33
12	1.26	0.07	1.12	1.19	1.26	1.33	1.4
13	1.27	0.08	1.11	1.19	1.27	1.34	1.42
14	1.33	0.07	1.19	1.26	1.33	1.4	1.47
15	1.37	0.06	1.25	1.31	1.37	1.42	1.48
16	1.37	0.07	1.24	1.31	1.37	1.44	1.51
17	1.39	0.06	1.27	1.33	1.39	1.44	1.5
18	1.4	0.05	1.3	1.35	1.4	1.45	1.51

### Statistical analysis and construction of reference curves

We characterized the sample by computing descriptive statistics, calculated SDS values, and excluded 29 TBS value outliers (SDS > 2 by age group). We performed a sensitivity analysis examining the impact of their inclusion on mean TBS values and confirmed that their removal did not significantly alter the results.

**Fig. 2:**
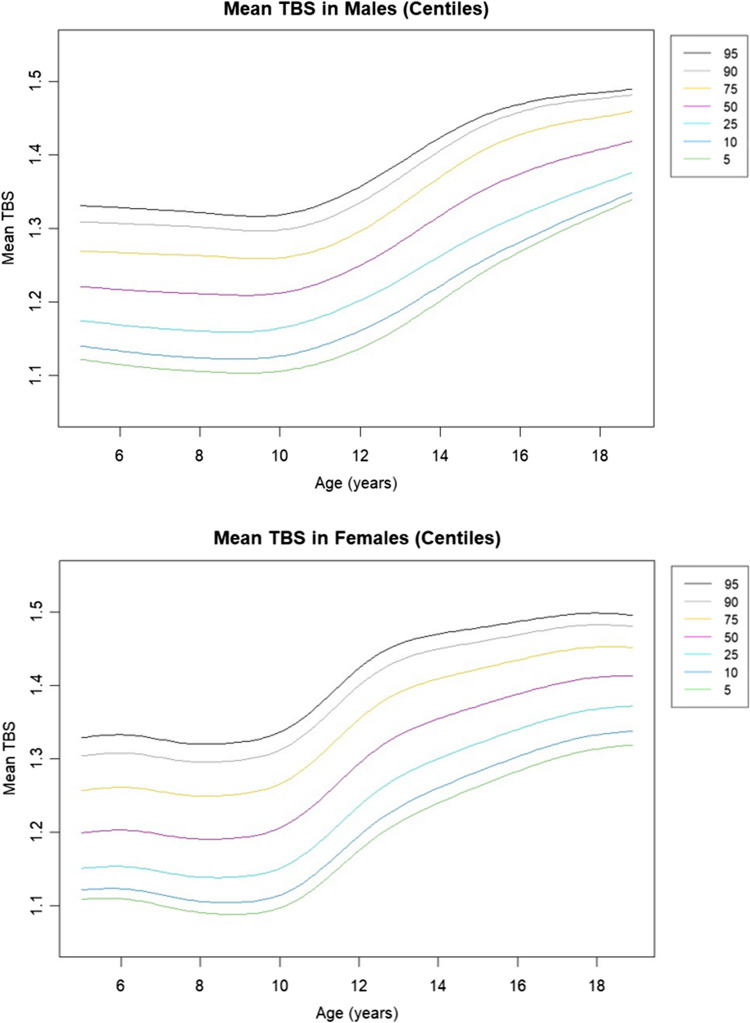
Age-related percentile curves for mean trabecular bone score (TBS) version 4.0 (core module version 19.4.0) in boys aged 5 to 18 years. Percentiles are displayed in legend. Lines represent smoothed percentiles fitted using the GAMLSS model

We constructed smoothened percentile growth curves using the Generalized Additive Models for Location, Scale, and Shape (GAMLSS) [20], applied through maximum likelihood estimation in R version 4.4.2, facilitated by R Studio. These GAMLSS models, advancing beyond the traditional LMS method [21], allowed us to effectively capture the nuanced, age-related trends in TBS values across our participant group, aged 5 to 18 years, using the GAMLSS model with the Box-Cox Power Exponential (BCPE) distribution given its flexibility for handling asymmetric data. The resulting percentile curves, depicting the 5th through 95th centiles for TBS, are illustrated in Fig. 2.

**Fig. 3:**
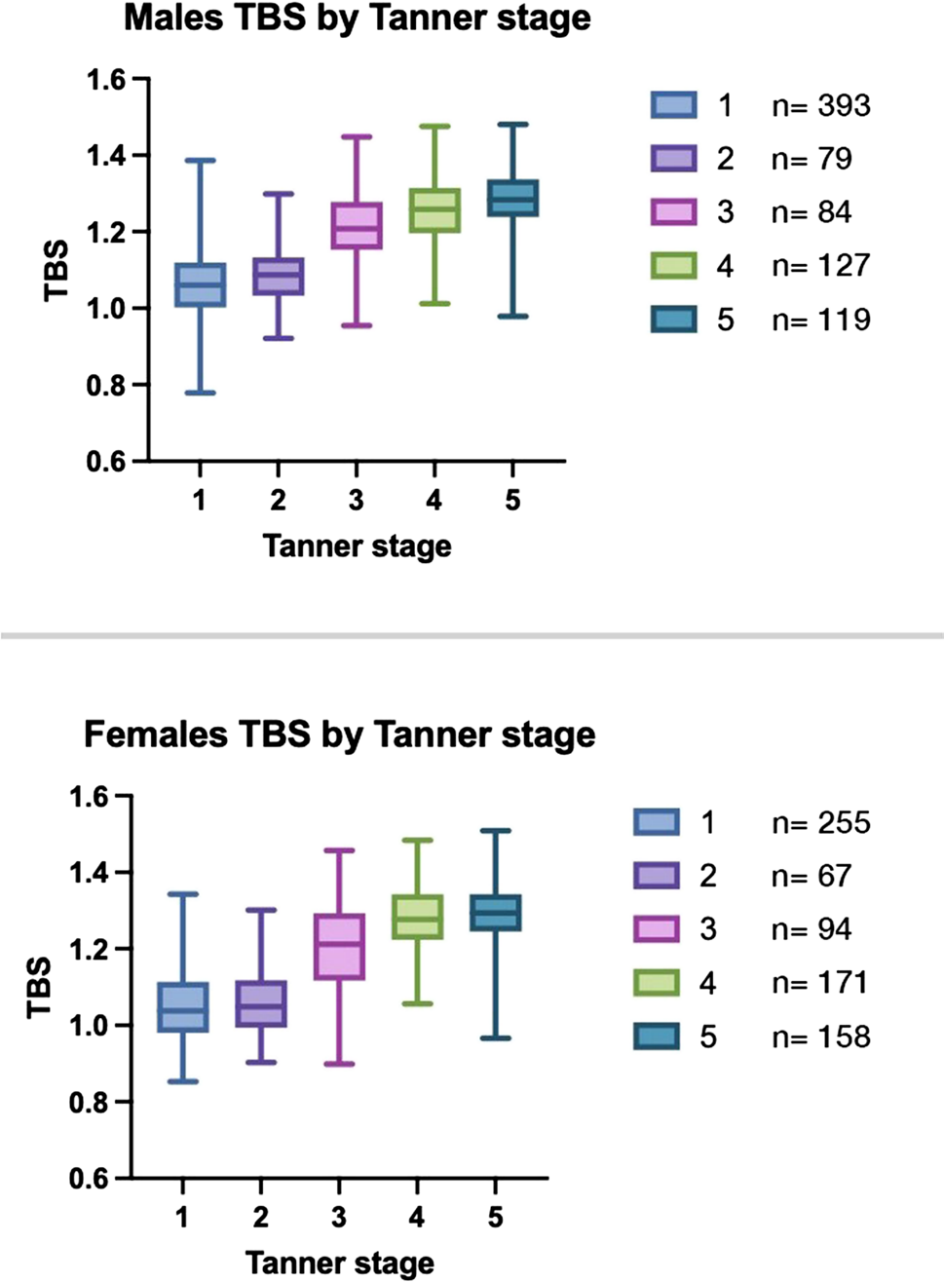
Boxplot representing TBS distributions by Tanner stage for males and females. In both sexes, TBS values increase progressively with pubertal development, with a marked rise from Tanner stages 2 to 4. The trend is more pronounced in females, consistent with earlier pubertal timing. Sample sizes for each stage are shown alongside the boxplots

Finally, we analyzed a subgroup of participants from whom information about their puberal Tanner stage had been obtained. Results are summarized in Table 3 and Fig. 3 shows the corresponding boxplot graph.

**Table 3:** Trabecular bone score (TBS) version 4.0 (core module version 19.4.0) reference values according to sex and pubertal stage

	Tanner stage				
	1	2	3	4	5
	255	67	94	171	158
Minimum	0.85	0.9	0.9	1.06	0.97
25% Percentile	0.98	0.99	1.12	1.22	1.25
Median	1.04	1.05	1.21	1.28=	1.29
75% Percentile	1.11	1.12	1.29	1.34	1.34
Maximum	1.34	1.3	1.46	1.48	1.51
Males	1	2	3	4	5
Number	393	79	84	127	119
Minimum	0.78	0.92	0.96	1.01	0.98
25% Percentile	1	1.03	1.15	1.2	1.24
Median	1.06	1.09	1.21	1.26	1.28
75% Percentile	1.12	1.13	1.28	1.32	1.34
Maximum	1.39	1.3	1.45	1.48	1.48

## Discussion

Our study is pioneering in analyzing TBS in a sample of 1533 healthy children and adolescents from Mexico City to provide reference values as an essential foundation for understanding bone health in the pediatric age. So far, TBS has been studied extensively in adults, demonstrating its value in assessing fracture risk and predicting osteoporosis independently of areal bone mineral density (aBMD). However, there is a notable paucity of studies on TBS in healthy children, with only a few exploratory studies focusing on children with specific medical conditions affecting bone health. Our study addresses the significant gap in pediatric bone health research by providing detailed TBS reference values for healthy Mexican children and adolescents. This is the first study using a Lunar/GE DXA scanner and the latest TBS iNsight beta software, version 4.0 (Core Module version 19.4.0), which accounts for abdominal soft tissue thickness and enhances the reliability of our measurements. The large sample size, the inclusion of carefully selected healthy children, and the rigorous statistical methods (GAMLSS) used for constructing growth curves contribute to the robustness of our findings.

One of the hallmark studies for the evaluation of bone health in children using DXA was the Bone Mineral Density in Childhood Study (BMDCS), which established that bone accrual follows nonlinear age trajectories, with BMC and aBMD increasing progressively, accelerating during puberty, and plateauing in late adolescence [22, 23]​. Such findings in BMC and aBMD were found in a similar pattern in Mexican children and adolescents, although with some differences in the previous study from our group. In the current paper, we similarly observed sex-specific pubertal changes in TBS, with a sharp increase in females from Tanner stage 2 to 3, aligning with their earlier growth spurt. While BMDCS introduced BMAD and height-adjusted aBMD (aBMDHAZ) to reduce stature-related biases [24], our study addresses body composition influences on TBS using the latest algorithm for soft tissue correction. By providing the first TBS reference curves for Mexican children and adolescents, we highlight the need for population-specific normative data. These findings suggest that TBS, alongside BMAD and aBMD, may enhance pediatric bone health assessment, particularly in tracking pubertal bone changes.

Establishing TBS reference values for Mexican children @provides important baseline data for future research evaluating the effects of diseases on bone health in children. Our findings indicate significant sexual dimorphism and age-related trends in TBS values: data curves from boys are different than those of girls, in which values increase earlier and in an increased tempo, compared to boys.

In girls, TBS increase takes place more steeply and starts from age 9 only to reach an apparent peak by age 16, while in boys it starts from ages 10–11 and the increase seems more gradual. However, in both sexes, the increase is most remarkable from Tanner stage 2 to 3. This has a biological sense since: [1] Tanner stage 3 corresponds with most intense phase of the growth spurt caused by a sharp rise in sex steroids and growth hormone and [2] Puberty starts on average 1 to 2 years earlier in girls than in boys and it presents in a shorter period on average [25]. Such rapid increase is also observed in BMAD curves, which accounts for the size of the lumbar vertebrae, but not in aBMD as is shown in data previously published by our group.

The trends in the data may be explained by the age of the onset of puberty and its related surge in the production of sexual steroids (estrogens and testosterone), which in turn impact trabecular bone [26]. However, not only the intensity of accretion is different, but so is the tempo. Bailey et al., using DXA measurements in a longitudinal study of pubertal girls and boys showed that changes of bone mineral content (BMC) in function of time present a peak BMC velocity (PBMCV) in average 18 months later in boys (average 13.44 years) than in girls (average 11.77 years) [27]. In our data, analyzing mean TBS by age group, boys seem to reach a “valley” from age 7 to 9, for a later increase of almost 26% by age 16. In girls, though, TBS values seem to reduce during childhood to a lower point by age 9; from there, the increase is up to 23% by age 18. Since this is one of the first studies to analyze TBS increase with age in the pediatric population, further cross-sectional and longitudinal studies are needed, including children, adolescents, young adults, and adults, to find patterns of increase and decline in TBS along the life course. This reinforces the importance of considering bone age and pubertal stages in pediatric bone health assessments [11].

Our study provides TBS reference curves for Mexican children and adolescents, and while direct comparisons are limited, trends observed in our data align with those reported in the USA by Kalkwarf et al. When we compared the trajectories of both populations, TBS values in both sexes are remarkably similar, except for the fact that average values are larger in the US sample (Supplementary graph 1). However, American data derived from five US centers were analyzed using an earlier version of the TBS software, which may account for variations in absolute values. These findings highlight the growing utility of TBS for assessing bone health in pediatric populations worldwide.

This is the second study reporting TBS reference data for a Latin American population and the first using Lunar/GE DXA. The Brazilian study by Fraga et al. used Hologic equipment, included a smaller sample size of 349 participants grouped into triennial age bands, and analyzed data with an older TBS software version (3.0.3.0), which is not optimized for pediatric use. Their results showed higher mean TBS values compared to our data and those from the USA, possibly reflecting methodological differences or population characteristics.

As DXA became widely available in the 1990 s, aBMD measurements across age groups revealed distinct patterns in bone dynamics, leading to the emergence of key concepts such as peak bone mass and the life-course trajectory of bone mineralization. These milestones have since become fundamental in understanding and assessing bone health. In contrast, comparable longitudinal data for TBS across the life course remains unavailable. While it is plausible that it follows a similar trajectory, including a potential “peak TBS,” this has yet to be documented.

In a previous work, we published TBS reference data for Mexican adults over 50 years of age [28]. In it, mean TBS for the younger age group (50–59 years) was 1.359 in women and 1.382 in men. Yet in the current study, 18-year-old participants in our study had mean TBS values of 1.404 for males and 1.413 for females, slightly higher compared to the adult means. First, it is important to note, that this analysis was conducted using an earlier software version. However, some other considerations should be noted. Improvements in childhood health indicators—such as nutrition, physical activity, and vaccination coverage—over recent decades may contribute to better bone microarchitecture in today’s adolescents. Socioeconomic development, particularly increased access to education and healthcare, could also support healthier growth trajectories. Additionally, regional differences in environmental exposures or lifestyle factors between our pediatric cohort and the adult sample may influence TBS values since the adult study included adults from four areas in Mexico, while our study only included Mexico City and it surrounding area. These factors highlight the importance of establishing age- and population-specific reference data for accurate interpretation of TBS. In addition, the lack of a continuous set of reference data from childhood through to older adulthood remains a gap in research on TBS.

However, our study also has limitations. The first is its cross-sectional design, which limits the ability to observe changes over time, which could provide deeper insights into the impact of puberty and other developmental factors on bone health. Longitudinal studies are needed to observe TBS changes over time and understand the long-term implications of pediatric TBS values on adult bone health. Further research is required to explore the underlying causes of the observed differences in TBS values between populations, such as genetic and environmental factors. As such, the reference ranges obtained may not apply to Mexican children living outside Mexico City metropolitan area.

The study addresses a significant gap in pediatric bone health research, particularly in a Latin American context, enhancing the understanding of TBS application across different populations. The data on TBS in children and adolescents may be highly valuable in decision-making. For pediatricians and pediatric subspecialists, TBS provides a deeper understanding of bone health that goes beyond traditional aBMD measurements. Unlike aBMD, TBS estimates bone microarchitecture, offering insights into bone quality and the structural integrity of trabecular bone. This is particularly important in children and adolescents, whose bones are developing rapidly and may present early signs of bone fragility that aBMD alone might not detect. Therefore, TBS provides an additional tool to aid in the evaluation of pediatric bone diseases and may be highly valuable in decision making, particularly in children with conditions like osteogenesis imperfecta, inflammatory bowel disease, or those undergoing treatments that affect bone density, such as systemic glucocorticoids.

Our study provides valuable reference curves for TBS in a Hispanic pediatric population, addressing an important gap in pediatric bone health research. While the cross-sectional design offers a comprehensive snapshot of TBS across age and pubertal stages, future longitudinal studies could provide deeper insights into the progression of TBS during development and its long-term implications for adult bone health. Additionally, further research is needed to explore the underlying genetic and environmental factors that may account for observed differences in TBS values between populations. These reference curves hold significant clinical relevance, as they can serve as a foundation to assess TBS in pediatric clinical populations, with potential uses in many patients with conditions affecting bone health.

## Conclusion

TBS holds a significant promise for enhancing our understanding and evaluation of bone health in children and adolescents. While it shows potential as a tool for guiding clinical interventions to mitigate fracture risks and prevent osteoporosis in later life, its integration into routine clinical practice awaits further validation. Comprehensive research is essential to elucidate TBS’s role in monitoring bone health across all stages of child development, paralleling the strides made with aBMD assessments in the early 1990 s that led to the concept of peak bone mass. The ISCD underscored this need in 2019, advocating for bone health evaluations that could inform interventions to reduce fracture risk among children and adolescents [29]. By integrating TBS into routine clinical practice, healthcare providers may better identify children at risk of bone fragility and intervene early.

Expanding TBS usage in pediatric care could significantly advance our ability to identify children at risk of bone fragility, whether due to inherent bone diseases or as side effects of treatments for other conditions, such as systemic glucocorticoids. However, the current scope of TBS research, limited to individuals up to 18 years of age, points to a gap in our understanding of TBS dynamics from late adolescence into early adulthood. Investigating TBS values in individuals aged 18 to 30 could offer invaluable insights into bone health progression, potentially mirroring the peak bone mass insights from BMD studies. Such endeavors are crucial for developing a continuous spectrum of TBS reference values, ultimately enhancing bone health assessments from childhood to adulthood.

## Supplementary Information

Below is the link to the electronic supplementary material.

ESM 1 (DOCX 371 KB)

ESM 2 (DOCX 15.6 KB)

## Data Availability

The data that support the findings of this study are available from the corresponding author upon reasonable request.
