# Supplementary material for: Reference curves for trabecular bone score adjusted for soft tissue thickness in children and adolescents from Mexico City
Source: Arch Osteoporos. 2025 Aug 6;20(1):110. doi: 10.1007/s11657-025-01595-4 (PMC12325490; doi:10.1007/s11657-025-01595-4)
Supplement: Supplementary file 1 — (DOCX 371 KB) [file 11657_2025_1595_MOESM1_ESM.docx]

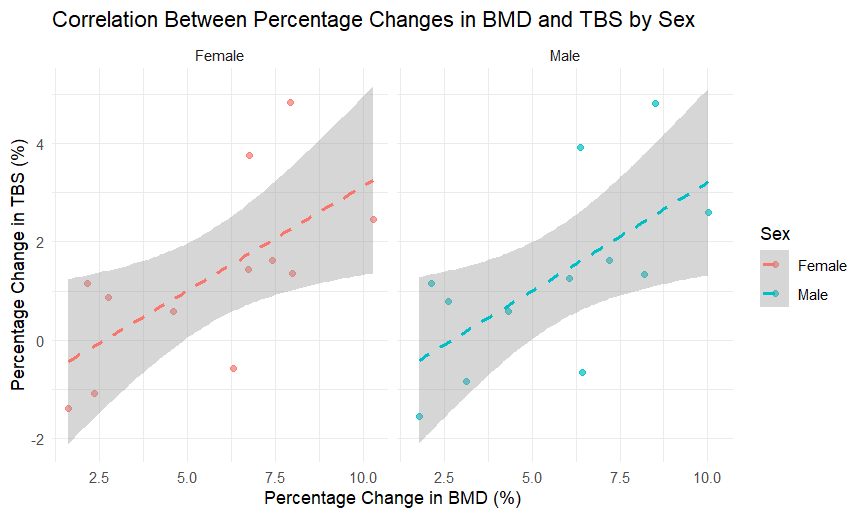


Supplementary Figure 1: The relationship between yearly percentage changes in areal bone mineral density (BMD) and trabecular bone score (TBS), analyzed separately by sex. A moderate positive correlation was observed for both males (r = 0.644) and females (r = 0.651) using Spearman’s rank correlation. These findings indicate that while BMD and TBS tend to increase in parallel during growth, they are not perfectly colinear, suggesting that TBS captures additional aspects of bone quality not reflected in BMD alone. The linear trend lines with confidence intervals further illustrate the consistency of this association across both sexes, supporting the complementary role of TBS in pediatric bone health assessment.


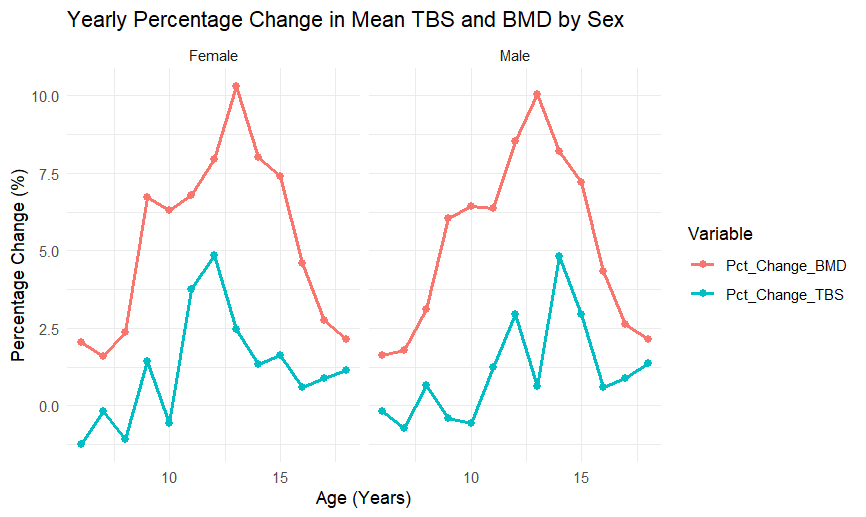


Supplementary Figure 2: Yearly percentage change in mean trabecular bone score (TBS) and areal bone mineral density (BMD) across ages 5 to 18, separated by sex. Both BMD and TBS show distinct patterns of bone accrual, with BMD exhibiting a sharper and more consistent rise during puberty, peaking around ages 12–13 in females and 13–14 in males, followed by a gradual decline in the rate of change. In contrast, TBS displays a more variable pattern, with smaller annual gains and more year-to-year fluctuation. These findings reflect the different biological processes captured by each measure—BMD representing mineral accumulation and TBS reflecting bone microarchitecture. The earlier peak in females aligns with known differences in pubertal timing, reinforcing the need for sex-specific reference data in pediatric bone health assessment.

Supplementary figure 3: Smoothed trajectories of mean trabecular bone score (TBS) by age between children from Mexico and the United States, stratified by sex. In both males and females, the overall shape of the TBS curve is similar across populations, with a slight decline in early childhood followed by a steep rise beginning near the onset of puberty. However, U.S. children show consistently higher mean TBS values across most ages, particularly after age 12. These differences may reflect population-specific influences such as body composition, nutrition, physical activity, or DXA machine calibration differences. Despite these offsets, the aligned pattern of growth reinforces the generalizability of TBS trajectories across populations, while underscoring the importance of establishing local reference curves for clinical interpretation.
