# Supplementary material for: Reference curves for trabecular bone score adjusted for soft tissue thickness in children and adolescents from Mexico City
Source: Arch Osteoporos. 2025 Aug 6;20(1):110. doi: 10.1007/s11657-025-01595-4 (PMC12325490; doi:10.1007/s11657-025-01595-4)
Supplement: Supplementary file 2 — (DOCX 15.6 KB) [file 11657_2025_1595_MOESM2_ESM.docx]

Supplementary table

TBS centiles

Female Centiles Male centiles

Age 3 5 10 25 50 75 90 95 97 Age 3 5 10 25 50 75 90 95 97

5 1.092 1.097 1.108 1.137 1.189 1.255 1.306 1.330 1.344 5 1.127 1.135 1.150 1.179 1.220 1.265 1.303 1.325 1.339

5.5 1.103 1.109 1.121 1.150 1.202 1.264 1.313 1.336 1.350 5.5 1.116 1.125 1.141 1.173 1.218 1.266 1.305 1.327 1.340

6 1.110 1.116 1.129 1.158 1.209 1.269 1.315 1.338 1.351 6 1.106 1.116 1.134 1.168 1.216 1.266 1.306 1.327 1.340

6.5 1.106 1.113 1.126 1.157 1.207 1.265 1.309 1.331 1.344 6.5 1.098 1.109 1.127 1.164 1.214 1.266 1.305 1.326 1.338

7 1.094 1.102 1.116 1.147 1.197 1.254 1.297 1.319 1.332 7 1.093 1.104 1.124 1.162 1.214 1.266 1.305 1.325 1.337

7.5 1.084 1.093 1.108 1.140 1.190 1.245 1.288 1.311 1.324 7.5 1.091 1.103 1.123 1.162 1.215 1.268 1.307 1.327 1.338

8 1.081 1.090 1.106 1.139 1.189 1.245 1.289 1.313 1.328 8 1.092 1.104 1.124 1.163 1.217 1.270 1.308 1.327 1.339

8.5 1.082 1.091 1.107 1.141 1.192 1.250 1.297 1.323 1.339 8.5 1.092 1.104 1.123 1.162 1.215 1.267 1.305 1.324 1.335

9 1.082 1.092 1.108 1.143 1.195 1.253 1.302 1.329 1.345 9 1.091 1.102 1.121 1.159 1.210 1.260 1.297 1.316 1.327

9.5 1.081 1.091 1.107 1.142 1.193 1.251 1.299 1.326 1.342 9.5 1.094 1.105 1.123 1.159 1.206 1.254 1.291 1.310 1.321

10 1.083 1.093 1.109 1.144 1.196 1.254 1.301 1.326 1.342 10 1.101 1.112 1.130 1.164 1.208 1.254 1.289 1.309 1.321

10.5 1.094 1.104 1.121 1.157 1.210 1.270 1.317 1.342 1.357 10.5 1.111 1.121 1.139 1.172 1.214 1.258 1.294 1.314 1.327

11 1.116 1.126 1.143 1.181 1.237 1.299 1.347 1.371 1.386 11 1.118 1.129 1.147 1.180 1.222 1.266 1.303 1.325 1.339

11.5 1.143 1.153 1.172 1.211 1.270 1.334 1.381 1.405 1.419 11.5 1.124 1.136 1.155 1.190 1.233 1.278 1.318 1.341 1.356

12 1.169 1.180 1.199 1.240 1.301 1.366 1.412 1.434 1.448 12 1.129 1.142 1.162 1.199 1.245 1.294 1.335 1.359 1.375

12.5 1.188 1.200 1.220 1.263 1.325 1.389 1.434 1.456 1.469 12.5 1.134 1.148 1.170 1.209 1.259 1.310 1.351 1.375 1.390

13 1.198 1.211 1.233 1.278 1.340 1.402 1.445 1.466 1.479 13 1.145 1.160 1.182 1.224 1.276 1.328 1.369 1.390 1.403

13.5 1.207 1.221 1.244 1.289 1.349 1.406 1.447 1.467 1.479 13.5 1.165 1.179 1.202 1.244 1.299 1.350 1.388 1.407 1.419

14 1.221 1.235 1.258 1.302 1.357 1.409 1.447 1.466 1.478 14 1.190 1.203 1.225 1.267 1.322 1.374 1.408 1.425 1.435

14.5 1.237 1.251 1.274 1.316 1.366 1.414 1.449 1.467 1.478 14.5 1.215 1.227 1.247 1.288 1.343 1.395 1.427 1.441 1.449

15 1.252 1.266 1.289 1.328 1.376 1.421 1.454 1.472 1.482 15 1.235 1.244 1.262 1.301 1.358 1.410 1.441 1.453 1.460

15.5 1.265 1.279 1.300 1.338 1.384 1.428 1.460 1.477 1.488 15.5 1.248 1.255 1.271 1.309 1.368 1.421 1.451 1.462 1.468

16 1.278 1.290 1.310 1.346 1.391 1.433 1.465 1.481 1.491 16 1.259 1.266 1.279 1.317 1.376 1.430 1.461 1.471 1.476

16.5 1.287 1.298 1.317 1.352 1.396 1.439 1.470 1.486 1.496 16.5 1.275 1.280 1.293 1.328 1.384 1.437 1.467 1.477 1.481

17 1.293 1.304 1.322 1.356 1.401 1.445 1.477 1.493 1.503 17 1.293 1.297 1.308 1.340 1.392 1.442 1.471 1.480 1.484

17.5 1.296 1.307 1.325 1.360 1.407 1.452 1.485 1.502 1.512 17.5 1.308 1.312 1.322 1.351 1.399 1.445 1.472 1.481 1.485

18 1.300 1.310 1.328 1.362 1.409 1.455 1.488 1.504 1.514 18 1.317 1.321 1.330 1.358 1.402 1.446 1.473 1.481 1.485
